# Tranexamic acid in a periarticular multimodal cocktail injection for blood management in total knee arthroplasty: a prospective randomized study

**DOI:** 10.1186/s12891-021-04551-8

**Published:** 2021-08-10

**Authors:** Kang-Il Kim, Jung-Kwon Bae, Jun-Ho Kim, Hyun-Gon Gwak, Sang Hak Lee

**Affiliations:** 1 Department of Orthopaedic Surgery, Kyung Hee University Hospital at Gangdong, 892 Dongnam-ro, Gangdong-gu, Seoul, 134-727 Republic of Korea; 2 Department of Orthopaedic Surgery, Kyung Hee University School of Medicine, Seoul, Korea

**Keywords:** Total knee arthroplasty, Tranexamic acid, Blood loss

## Abstract

**Background:**

This study aimed to assess the efficacy of tranexamic acid (TXA) mixed in a periarticular multimodal cocktail (PAMC) as a topical administration and to determine whether combined use of intravenous and topical administration is more effective than a single administration of TXA.

**Methods:**

A total of 240 patients who underwent primary total knee arthroplasty (TKA) was enrolled for this prospective randomized controlled study. Patients were divided into three groups of 80 patients each. Baseline data were comparable for all groups. Average follow-up was 18.7 months. Group 1 consisted of patients who received intravenous (IV) TXA, Group 2 patients were those who received TXA in a PAMC injection for topical administration, and Group 3 consisted of patients who received a combination of both intravenous and topical administration of TXA. Primary outcomes were postoperative hemoglobin drop and amount of suction drainage. Secondary outcomes were estimated blood loss (EBL), postoperative transfusion rate, and complications.

**Results:**

The mean postoperative hemoglobin drop was significantly lower in Group 3 (2.13 ± 0.77 g/dL, p=0.004), and there was no difference between Group 1 and Group 2 (2.56 ± 1.07 g/dL vs 2.55 ± 0.86 g/dL, p=0.999). The mean drainage amount was significantly lower in Group 3 (326.58 ± 57.55 ml, p<0.001), and there was no difference between Group 1 and Group 2 (367.93 ± 87.26 ml vs 397.66 ± 104.10 ml, p=0.072). Similarly, the mean EBL was significantly lower in Group 3 (p=0.003), and there was no significant difference between Group 1 and Group 2 (p=0.992). There were no significant differences in requirement for postoperative transfusion rate or incidence of complications among the three groups.

**Conclusion:**

TXA mixed in a PAMC injection showed a similar effect to IV administration of TXA following TKA. Furthermore, combined use of both IV and PAMC injection provided better perioperative bleeding control with similar safety in patients without relevant comorbidities.

**Trial registration:**

WHO ICTRP identifier KCT0005703. Retrospectively registered: 12/24/2020

**Supplementary Information:**

The online version contains supplementary material available at 10.1186/s12891-021-04551-8.

## Background

Tranexamic acid (TXA) is an anti-fibrinolytic agent that inhibits the conversion of plasminogen to plasmin and also acts as a plasmin inhibitor [1]. Therefore, TXA administration can decrease bleeding by acting on the fibrinolytic system [2]. Many studies have reported that intravenous (IV) administration of TXA significantly reduces postoperative blood loss and need for transfusion without related complications in patients undergoing total knee arthroplasty (TKA) [3–7]. However, since thromboembolic events present a concern in TXA use in certain patient subsets (recent cardiovascular disease and strong history of VTE) [8–10], topical application of TXA has been adopted [11, 12]. In theory, patients at risk associated with use of IV TXA may tolerate topical administration without increased risk of systemic adverse events due to the presumed delay in systemic absorption [11]. Compared with IV TXA, intra-articular (IA) application of TXA as a topical injection has shown similar efficacy in reducing both blood loss and transfusion rate in primary TKA [13–15]. However, the methods of topical administration of TXA are variable and somewhat complicated [11, 13, 16, 17]. Another approach is periarticular injection of TXA that acts directly on the injured tissue [18–20]. Periarticular multimodal cocktail (PAMC) injection has been commonly used to control postoperative pain in TKA [21–23]. However, there is a paucity of studies regarding PAMC injection, including studies on use of TXA for control of perioperative pain and bleeding [24]. Research has been limited to a retrospective study with small sample size [24]. We assumed that TXA mixed in a PAMC injection might be more simplified method among existing topical TXA methods and have a similar effect to IV TXA administration. Thus, we hypothesized that combined use of topical and IV TXA would provide better postoperative bleeding control with similar safety. In this prospective randomized study, postoperative hemoglobin drop and total drainage amount for the efficacy of TXA as a primary outcome were assessed.

## Methods

### Inclusion and exclusion criteria

**Fig. 1:**
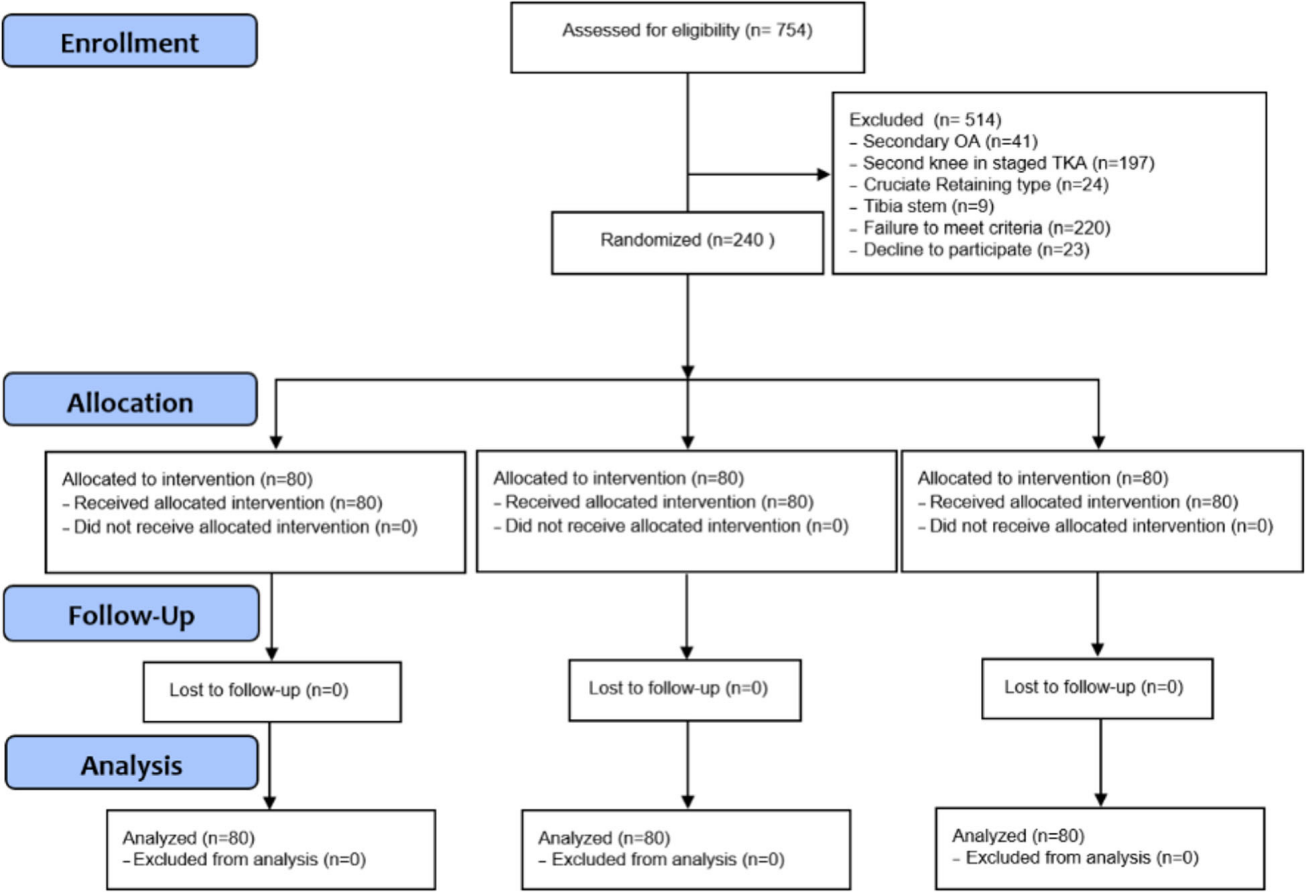
CONSORT flow diagram for this clinical trial

Written informed consent was obtained prospectively from all patients prior to surgery, and the study protocol was approved by the institutional review board (KHNMC 2016-12-005). The authors confirm that all ongoing and related trials for this drug/intervention are registered and adheres to CONSORT guidelines. The inclusion criteria was primary osteoarthritis (OA) patients who underwent unilateral TKA using a cemented, posterior-stabilized prosthesis. Patients who took antiplatelet or anticoagulants were instructed to discontinue 5 days prior to surgery. Platelet responses to preoperative nonsteroidal anti-inflammatory (NSAID) therapy were not considered. Patients with secondary OA were not eligible for inclusion in this study; those with a second knee in staged bilateral TKA, cruciate-retaining type of prosthesis, or a tibia stem fixation for primary TKA were also excluded to reduce bias. Exclusion criteria also included presence of major comorbidities such as severe ischemic heart disease, severe pulmonary disease, severe renal insufficiency, hepatic failure, coagulopathy, and a history of arterial or venous thromboembolic disease (cerebrovascular accident, deep vein thrombosis, or pulmonary thromboembolism). The present study prospectively designed and collected clinical data from patients who underwent TKA at our institute from May 2017 to December 2019. Finally, a total of 240 patients was enrolled in the study (Fig. 1). Average follow-up was 18.7 months and there was no lost follow-up at 6 months.

### Randomization and blinding

After consenting to participate in the study on the morning of surgery, patients were randomly assigned prior to the surgery through a central computer-derived randomization table. In the operation room, a scrub nurse prepared the study medication after an investigator provided the intraoperative prescription based on the patient’s allocations.

### Interventions

Group 1 (n = 80) consisted of patients who received a dose of 15 mg/kg IV TXA (tranexamic acid inj.: Shin Poong Pharm. Co.,Ltd., Seoul, Korea) with 100 ml normal saline twice in the TKA perioperative period [25]. The first infusion was administered before tourniquet release in the operating room. The second infusion was administered 6 hours after surgery while the patient was on the ward [26]. PAMC without TXA was directly injected into the area around the medial, lateral, anterior and posterior capsule; the quadriceps muscle tendon; and the infrapatellar fat pad just prior to cementation. Group 2 (n = 80) patients received 1 g of TXA [27] mixed with PAMC. The PAMC contained 150 mg ropivacaine, 0.3 mg epinephrine, 45 mg ketorolac, 40 mg triamcinolone, 5 mg morphine, 1 g cefotiam, and 60 ml normal saline [21, 22, 28]. TXA was directly injected in the same manner as PAMC administration in group 1. Group 3 (n = 80) patients received combination therapy, both IV and PAMC injections. A dose of 15 mg/kg IV TXA with 100 ml normal saline was administered twice in the same manner as in Group 1 patients. In addition, 1 g of TXA mixed in PAMC was given in the same manner as in Group 2 [14, 29].

### Surgical methods and postoperative treatment

All TKAs were performed by a senior surgeon (KIK) in a single institute. A pneumatic tourniquet was applied during the operation and deflated before wound closure. Cemented components were used in all cases. The prosthesis used was the posterior-stabilized Attune Knee System (Depuy Synthes, Warsaw, IN) in 144 knees, the Truliant Knee System (Exactech, Gainesville, FL, USA) in 56 knees, the Triathlon Knee System (Stryker, Mahwah, NJ, USA) in 40 knees. The patella was resurfaced in all cases. Perioperative TXA administration was applied according to the protocol determined for each group. A suction drain was placed into the knee joint and opened without clamping. The trigger for transfusion was a postoperative hemoglobin level lower than 8.0 g/dL with clinical symptoms of anemia any time during the postoperative period [30]. An elastic stocking and intermittent pneumatic compression device were applied after surgery. All patients received the same postoperative rehabilitation. This included range of motion, calf pump exercises, straight leg raising, and bedside continuous passive mobilization on postoperative day 1. The suction drain was removed 24 hours postoperatively, and patients were allowed to walk as tolerated.

### Outcome measures

The demographic parameters were compared among the groups were sex, site, age, body mass index (BMI) and American Society of Anesthesiologists (ASA) classification [31] (Table 1). The hematologic parameters compared among three groups were hemoglobin level, which was monitored preoperatively and at the postoperative first, second, and fifth days; total amount of suction drainage; estimated blood loss (EBL); and need for transfusion and related complications. The postoperative hemoglobin drop was calculated when hemoglobin level was reduced to the maximum after surgery. EBL was calculated by the formula of Nadler et al. [32] and Good et al. [33] using the maximum drop in hemoglobin level after surgery, adjusted for height and weight of the patient. Primary outcomes were hemoglobin drop and total drainage amount. Secondary outcomes were EBL, transfusion rate, and complications. Clinical complications and readmission until postoperative 6 month after discharge were recorded and compared among the groups. As clinical outcomes, hemarthrosis, wound dehiscence, periprosthetic joint infection (PJI), deep vein thrombosis (DVT) and stiffness were recorded. Hemarthrosis was evaluated patellar circumference [34]. Acute postoperative PJI was diagnosed based on ICM-modified definition in 2013 [35]. Preoperative doppler sonography was performed prior to TKA for DVT evaluation. The incidence of venous thromboembolism (DVT and symptomatic pulmonary embolism) was evaluated from CT venography which performed at postoperative day 5. Any short of breath, chest pain or blood-streaked sputum suggestive of pulmonary embolism was evaluated by PTE CT [26]. Stiffness is defined as using a cut-off of about 95 degrees of flexion that prevents the patient from doing most of activity [36].

**Table 1:** Patient demographics

	IV TXA (*n*=80)	PAMC TXA (*n*=80)	Combined (*n*=80)
Sex, male:female	13 : 67	13 : 67	11 : 69
Site, right:left	38 : 42	41: 39	35 : 45
Mean age, years	72.08 ± 4.63	72.58 ± 5.04	73.35 ± 4.16
Height, cm	155.82 ± 7.00	156.03 ± 7.06	155.41 ± 6.58
Weight, kg	63.96 ± 8.32	64.57 ± 8.88	62.89 ± 7.97
BMI, kg/m2	26.36 ± 3.24	26.54 ± 3.49	26.05 ± 3.16
Tourniquet time, min	64.31± 6.63	63.54± 5.42	63.71± 5.13
ASA classification			
I	20	23	26
II	51	46	48
III	9	11	6

*BMI* body mass index, *ASA* American society of anesthesiologists

### Sample size and statistical methods

Power analysis and sample size calculation were performed using PASS 2015 (NCSS LLC, Kaysville, Utah) software and R version 3.5.1 (R Foundation for Statistical Computing, Vienna, Austria) using the statistical test for One-way analysis of variance (ANOVA). The sample size was evaluated based on the measured primary outcome and calculated to be 78 knees in each group assuming 80% power, an alpha error = 0.05, and a dropout rate of 5%. A difference of 21% in Hb drop was chosen based on calculations of effect sizes from values of postoperative hemoglobin reported in randomized controlled trials of topical TXA use in TKA published at the time of study design [12]. Accounting for potential exclusions, 80 knees were included in each group. For continuous, normally distributed data (e.g., total blood loss and hemoglobin level), ANOVA was performed, followed by Tukey’s post-hoc correction. For categorical data (e.g., transfusion rate and VTE), these variables were analyzed using the chi-square or Fisher's exact test. The SPSS 24.0 (IBM Corp, Armonk, NY) program was used for statistical analysis with a significance threshold of *p* < 0.05.

## Results

There were no significant differences in hemoglobin level preoperatively or at 1 to 5 days postoperatively among the three groups (Table 2). Mean postoperative hemoglobin drop was significantly lower in Group 3 (2.13 ± 0.77 g/dL, p = 0.004) (Table 3). There was no significant difference between Groups 1 and 2 in postoperative hemoglobin drop (2.56 ± 1.07 g/dL vs 2.55 ± 0.86 g/dL, p=0.999). The mean drainage amount was lowest in Group 3 (326.58 ± 57.55 ml, p < 0.001), and there were no significant differences between Groups 1 and 2 in total drainage amount (367.93 ± 87.26 ml vs 397.66 ± 104.10 ml, p=0.072) (Table 4). Similarly, the mean EBL was lowest in Group 3 (611.97 ± 227.91 ml, p = 0.003), but there were no significant differences between Groups 1 and 2 (733.11 ± 296.43 vs 737.92 ± 250.94, p=0.992) (Table 5). One patient in each of Groups 1 and 2 required postoperative transfusion (Additional file 1: Table 1). Symptomatic pulmonary thromboembolic manifestation was not observed in any patients, and one proximal deep vein thrombosis occurred in a Group 3 patient. Clinical complication and readmission until postoperative 6 month did not show any significant differences among the groups. The readmission in the 6 months after TKA occurred one case in Group 1 and the reason for readmission were stiffness. Hemarthrosis occurred two cases in Group 2 and one case in Group 3. There was no PJI or any other significant complications.

**Table 2:** Hemoglobin change after TKA

	IV TXA (*n*=80)	PAMC TXA (*n*=80)	Combined (*n*=80)	*P* value ^a^
Preoperative Hb (g/dL)	12.93 ± 1.01	12.78 ± 1.03	12.74 ± 1.04	0.458
Postoperative day 1	11.42 ± 1.22	11.03 ± 1.05	11.35 ± 1.17	0.070
Postoperative day 2	10.38 ± 1.25	10.23 ± 0.94	10.61 ± 0.95	0.074
Postoperative day 5	10.89 ± 0.73	10.63 ± 0.94	10.77 ± 0.86	0.153

*TKA* total knee arthroplasty, *IV* intravenous, *PAMC* peri-articular multimodal cocktail, TXA tranexamic acid, Hb hemoglobin

^a^ Using one-way ANOVA for comparison among groups

**Table 3:** Postoperative hemoglobin drop compared among 3 groups

	IV TXA (*n*=80)	PAMC TXA (*n*=80)	Combined (*n*=80)	*P* value ^a^
Hb drop (g/dL)	2.56 ± 1.07	2.55 ± 0.86	2.13 ± 0.77	0.004
Post hoc test ^b^				
IV TXA	-	0.999 ^c^	0.010 ^d^	
PAMC TXA	0.999 ^c^	-	0.011 ^e^	
Combined	0.010 ^d^	0.011 ^e^	-	

*IV* intravenous, *PAMC* peri-articular multimodal cocktail, *TXA* tranexamic acid, *Hb* hemoglobin

^a^ Using one-way ANOVA for comparison among groups

^b^ Using Turkey’s test for pair wise comparison

^c^ group 1 vs group 2

^d^ group 1 vs group 3

^e^ group 2 vs group 3

**Table 4:** Total drainage amount compared among 3 groups

	IV TXA (*n*=80)	PAMC TXA (*n*=80)	Combined (*n*=80)	*P* value ^a^
Total drainage amount (ml)	367.93 ± 87.26	397.66 ± 104.10	326.58 ± 57.55	<0.001
Post hoc test ^b^				
IV TXA	-	0.072 ^c^	0.007 ^d^	
PAMC TXA	0.072 ^c^	-	<0.001 ^e^	
combined	0.007 ^d^	< 0.001 ^e^	-	

*IV* intravenous, *PAMC* peri-articular multimodal cocktail, *TXA* tranexamic acid

^a^ Using one-way ANOVA for comparison among groups

^b^ Using Turkey’s test for pair wise comparison

^c^ group 1 vs group 2

^d^ group 1 vs group 3

^e^ group 2 vs group 3

**Table 5:** Estimated blood loss compared among 3 groups

	IV TXA (*n*=80)	PAMC TXA (*n*=80)	Combined (*n*=80)	*P* value ^a^
Estimated blood loss (ml)	733.11 ± 296.43	737.92 ± 250.94	611.97 ± 227.91	0.003
Post hoc test ^b^				
IV TXA	-	0.992 ^c^	0.010 ^d^	
PAMC TXA	0.992 ^c^	-	0.007 ^e^	
combined	0.010 ^d^	0.007 ^e^	-	

*IV* intravenous, *PAMC* peri-articular multimodal cocktail, *TXA* tranexamic acid

^a^ P value is for one-way ANOVA was use to evaluate three groups

^b^ Using Turkey’s test for pair wise comparison

^c^ group 1 vs group 2

^d^ group 1 vs group 3

^e^ group 2 vs group 3

## Discussion

The most important finding of the present study is that TXA mixed in a PAMC injection as a simple topical administration showed a similar effect to IV administration of TXA following TKA. Moreover, combined use of IV and topical TXA injection showed significantly decreased postoperative hemoglobin drop, drainage amount, and EBL compared to a single administration of TXA and did not increase hematologic complications.

Several meta-analyses confirmed that IV administration of TXA reduces postoperative bleeding and need for transfusion [3, 4]. Though there has not been obvious evidence of thromboembolic complications after systemic use of TXA [8–10, 37, 38], topical application of TXA has been introduced to reduce this complication [11, 12]. Several studies reported that IV and topical injection of TXA showed similar effectiveness in reducing blood loss and transfusion following TKA [13, 14]. However, the methods of topical application of TXA are somewhat variable, and there seems to be no consensus concerning the most appropriate method [11, 13, 16, 17]. For topical administration, TXA may be applied by soaking the soft tissue in the operating field after prosthesis implantation, followed by washing [11, 16] or may be retrograde administered into the intraarticular area through a suction drain after skin closure (clamping method) and drain it out through the drainage tube [13, 17]. However, these procedures are not routine process in TKA and may increase the chance of periprosthetic joint infection by elongating operation time, exposing soft tissue by air during soaking, or retrograde TXA injection from out-side to the joint through a drainage tube after closure [39, 40]. Direct periarticular injection is an alternative route for topical administration of TXA [19, 20, 24]. Periarticular direct injection of TXA also has advantages such as allowing the surgeon to target areas those are vulnerable to postoperative bleeding [18, 23]. Meanwhile, classical PAMC injection has been widely used in TKA for perioperative multi-modal pain control [20–22]. Since PAMC injection is a safe and simple procedure that does not require additional time or a special technique, it would simplify the way of topical TXA administration and thus we designed a PAMC injection containing TXA for topical administration. Although, topical and IV TXA are off-label use in our country, the efficacy and safety of periarticular injection of TXA mixed in a PAMC have not been reported in a study using prospective design and adequate sample size (Additional file 2: Table 2). Finally, we confirmed that PAMC injection containing TXA reduces postoperative blood loss after TKA, similar to IV administration of TXA in this prospective randomized study.

There has been still controversy whether combined use of IV and topical application of TXA would have more effective regarding perioperative bleeding control without safety issue compared to single administration methods of TXA [41, 42]. The current study demonstrated that the combination of the two methods significantly reduces perioperative blood loss. In this regard, the combined method is superior to IV or topical administration alone. Moreover, there were no significant differences in adverse events among the three groups. Therefore, we consider that, along with the systemic effect of IV TXA, periarticular topical administration of TXA can inhibit local activation of fibrinolysis and may reduce time to vascular occlusion [43]. The combined method also has the advantage of limiting local blood loss [13]. Nielsen et al. [44] reported that combined administration of IV and IA TXA resulted in a clinically relevant reduction in blood loss of 37% compared with IV TXA alone both at 24 hours postoperatively and on postoperative day 2. Our findings are consistent with a meta-analysis of randomized controlled trials [45] and indicate that combined use of systemic and periarticular application of TXA in TKA significantly reduces postoperative hemoglobin drop and drainage volume without increasing thromboembolic complications. Based on these findings, TXA mixed in a PAMC for topical administration would be an alternative to IV administration of TXA in terms of perioperative bleeding control in patients with TKA. Furthermore, TXA mixed in a PAMC with IV administration of TXA may produce synergic effect with safety.

There are some limitations in this study. First, we did not have a no-TXA group as a control. However, the effectiveness of TXA in perioperative blood management during TKA has been proven in many studies. Therefore, there was no need for a separate control group, and we designed the IV TXA group as a control. Additionally, this study specifically focused primarily on hemoglobin levels, drain amount and EBL but did not compare functional outcomes and effects of the three methods of TXA administration. Second, we did not use the same prosthesis in all patients. However, we used posterior-stabilized knee design in all patients with patellar resurfacing to reduce influence on blood loss. Moreover, all operations were performed by a single senior surgeon. Therefore, the component bias would be minimal. Third, patients with severe comorbidities were excluded and relatively healthy patients were included in the current study. This healthy study population might provoke a concern of representing the majority of TKA patients regarding application of TXA. However, we performed randomized controlled trial to avoid selection bias and the exclusion criteria seemed to be reasonable considering potential adverse effect of TXA. Thus, results of the present study may be applicable to the majority of TKA patients. Fourth, this study didn’t compare with groups regarding the prevalence of hematologic complications. However, this is a randomized trial and it is not appropriate to compare groups at baseline and any differences that occur would be by chance.

## Conclusions

TXA mixed in a PAMC injection showed a similar effect to IV administration of TXA following TKA. Furthermore, combined use of both IV and PAMC injection provided better perioperative bleeding control with similar safety in patients without relevant comorbidities.

## Supplementary Information

**Additional file 1: Table 1**. Perioperative transfusion & complication.

**Additional file 2: Table 2**. Recent studies comparing peri-articular injection of tranexamic acid with other modalities.

## Data Availability

The datasets generated and/or analyzed during the current study are not publicly available due to participants did not consent to public data release of their data but are available from the corresponding author on reasonable request.

## Abbreviations

TXA: Tranexamic acid
TKA: Total knee arthroplasty
VTE: Venous thromboembolic event
PAMC: Periarticular multimodal cocktail
EBL: Estimated blood loss
BMI: Body mass index
