# Supplementary material for: Tranexamic acid in a periarticular multimodal cocktail injection for blood management in total knee arthroplasty: a prospective randomized study
Source: BMC Musculoskelet Disord. 2021 Aug 10;22:675. doi: 10.1186/s12891-021-04551-8 (PMC8356435; doi:10.1186/s12891-021-04551-8)
Supplement: Supplementary file 1 — Additional file 1: Table 1. Perioperative transfusion & complication. [file 12891_2021_4551_MOESM1_ESM.docx]

***Appendix table 1*** Perioperative transfusion & complication

|  | IV TXA  (n=80) | PAMC TXA  (n=80) | Combined  (n=80) | P value |
| --- | --- | --- | --- | --- |
| Transfusion | 1/80 | 1/80 | 0/80 | 0.776 |
| Total DVT | 5/80 | 4/80 | 5/80 | 1 |
| Proximal DVT | 0/80 | 0/80 | 1/80 | 1 |
| Symptomatic PTE | 0/80 | 0/80 | 0/80 | 1 |
| Infection | 0/80 | 0/80 | 0/80 | 1 |
| Wound problem | 0/80 | 0/80 | 1/80 | 1 |

IV intravenous, PAMC peri-articular multimodal cocktail, TXA tranexamic acid, DVT deep vein thrombosis, PTE pulmonary thromboembolic manifestation
