# Supplementary material for: Tranexamic acid in a periarticular multimodal cocktail injection for blood management in total knee arthroplasty: a prospective randomized study
Source: BMC Musculoskelet Disord. 2021 Aug 10;22:675. doi: 10.1186/s12891-021-04551-8 (PMC8356435; doi:10.1186/s12891-021-04551-8)
Supplement: Supplementary file 2 — Additional file 2: Table 2. Recent studies comparing peri-articular injection of tranexamic acid with other modalities. [file 12891_2021_4551_MOESM2_ESM.docx]

***Appendix table 2*** Recent studies comparing peri-articular injection of tranexamic acid with other modalities

| Study | Year | Study type | Sample size, n | Comparison | Main findings |
| --- | --- | --- | --- | --- | --- |
| Besiris et al. | 2020 | RCS | 66 | PAI vs IAI | Significantly lower transfusion rate in PAI TXA than in IAI TXA. |
| Mao et al. | 2016 | RCS | 126 | PAI vs IAI vs Placebo | Comparable results in blood loss, Hb drop, and blood transfusion rate. |
| Pinsornsak et al. | 2016 | RCT | 60 | PAI vs IV | Comparable results in blood loss, Hb drop, and blood transfusion rate. |
| Yozawa et al. | 2018 | RCS | 88 | PAI vs Placebo | Significantly lower Hb drop and blood loss in PAI TXA group. |
| Zhang et al. | 2019 | RCT | 218 | PAI vs IAI vs PAI+IAI vs Placebo | Significantly lower blood loss and blood transfusion rate in PAI + IAI TXA group. |
| Current study | | RCT | 240 | PAMC vs IV vs PAMC+IV | Significantly lower Hb drop and blood loss in PAMC+IV TXA group. Comparable results in Hb drop and blood loss between PAMC TXA and IV TXA. |

PAI peri-articular injection, IAI intra-articular injection, IV intravenous, PAMC peri-articular multimodal cocktail, Hb hemoglobin, TXA tranexamic acid, RCT randomized controlled trial, RCS retrospective comparative study
